# Periodic Fever, Aphthous Stomatitis, Pharyngitis, and Cervical Adenitis (PFAPA) Syndrome: An Updated Review with Current Insights into Pathogenesis and Therapeutic Strategies

**DOI:** 10.3390/children13091167

**Published:** 2026-08-29

**Authors:** Abobakr Abdelgalil, Doaa Mosad Mosa, Ghaidaa Faisal Albaz, Konooz Fahad Faisal, Ali Sobh, Mohamed Hesham Mashali

**Affiliations:** 1 Department of Pediatrics, Faculty of Medicine, Cairo University, Cairo 12613, Egypt; mhmdmashaly@gmail.com; 2 Department of Rheumatology, Rehabilitation, and Physical Medicine, Faculty of Medicine, Mansoura University Hospital, Mansoura University, Mansoura 35516, Egypt; doaamosad@mans.edu.eg; 3 Department of Pediatrics, King Abdulaziz University Hospital, Jeddah 21589, Saudi Arabia; galbaz97@gmail.com (G.F.A.); konoozfaisal@gmail.com (K.F.F.); 4 Department of Pediatrics, Faculty of Medicine, Mansoura University Children’s Hospital, Mansoura University, Mansoura 35516, Egypt; ali.sobh@mans.edu.eg

**Keywords:** PFAPA, monogenic autoinflammatory disorders, children, genetic

## Abstract

**Highlights:**

**What are the main findings?**

• PFAPA is the most common autoinflammatory syndrome in children, with evolving evidence indicating a multifactorial pathogenesis involving genetic susceptibility, innate immune dysregulation, and environmental triggers.
• Recent advances have refined the understanding of PFAPA diagnosis, highlighted its overlap with monogenic autoinflammatory diseases, and expanded therapeutic options beyond corticosteroids and tonsillectomy, including biologic agents for selected patients.

**What are the implications of the main findings?**

• Improved recognition of PFAPA and differentiation from infections and hereditary autoinflammatory disorders are essential to prevent unnecessary investigations, antibiotic use, and delays in diagnosis.
• Emerging insights into PFAPA pathogenesis and its relationship with the autoinflammatory disease spectrum may facilitate more personalized diagnostic and therapeutic approaches while guiding future research.

**Abstract:**

**Background/Objectives**: Periodic fever, aphthous stomatitis, pharyngitis, and cervical adenitis (PFAPA) syndrome is the most frequently encountered periodic fever syndrome in pediatric populations. It is characterized by recurrent, stereotyped episodes of fever accompanied by pharyngitis, cervical lymphadenopathy, and aphthous stomatitis, sometimes with other additional nonspecific symptoms. Episodes usually last 3–7 days and recur at regular intervals of approximately 2–8 weeks. The etiopathogenesis of PFAPA remains incompletely understood. Accumulating evidence supports a central role of immune dysregulation with multiple genetic variants that contribute to disease susceptibility. Diagnosis is primarily based on clinical evaluation, although targeted genetic testing may be considered in selected cases particularly when overlapping with other monogenic autoinflammatory disorders. Management of PFAPA aims to decrease the frequency and intensity of febrile episodes. Acute flares are managed with antipyretics, corticosteroids, or, in selected cases, anti-interleukin-1 (IL-1) therapy, while preventive strategies include colchicine, cimetidine, or tonsillectomy. Adjunctive approaches such as vitamin D supplementation, probiotics, and other immunomodulatory interventions have also been investigated. This updated review summarizes current insights into the pathogenesis and therapeutic approaches of PFAPA. **Methods**: This narrative review was conducted using PubMed, and Scopus to identify English-language publications from 2010 to 2026 addressing PFAPA. **Conclusions**: Pediatricians should consider PFAPA in children with recurrent fevers and oropharyngeal symptoms, especially when standard treatments fail, to ensure proper management and avoid unnecessary antibiotic use.

## 1. Introduction

Periodic fever, aphthous stomatitis, pharyngitis, and cervical adenitis (PFAPA) syndrome is the most frequently encountered periodic fever syndrome in non-Mediterranean children and the second most common one in Mediterranean ones after familial Mediterranean fever (FMF). PFAPA is classified as an autoinflammatory disorder and is characterized by stereotyped regularly recurring episodes of fever, pharyngitis, tender cervical adenopathy and aphthous stomatitis. Additionally, the episodes may be associated with non-specific symptoms such as abdominal pain, vomiting, diarrhea, headache, arthralgia, myalgia, and fatigue. Inflammatory markers are markedly elevated during the febrile flares and return to normal between the episodes [1,2]. Since its initial description by Marshall et al. in 1987 [3], several refinements of diagnostic and classification criteria were published; Modified Marshall’s [4], Cantarini et al. [5], Vanoni et al. [6], and New Euro fever/PRINTO [7].

A Norwegian population-based study reported an annual PFAPA incidence of 2.3 per 10,000 children aged ≤5 years, although comparable epidemiological data from other populations may vary, with a slight male predominance [8]. Disease onset is usually in early childhood, and most pediatric cases are benign, with spontaneous remission around puberty years. Nonetheless, persistent illness and adult-onset PFAPA have been reported. Furthermore, recurrences have been documented following apparent resolution, broadening its clinical spectrum. The actual incidence is likely underestimated as cases may be readily misclassified as upper respiratory tract infections, other periodic fever or cyclic neutropenia. Febrile episodes typically last 3–7 days and occur at predictable regular intervals of 2–8 weeks and children are asymptomatic between the episodes, and they have normal growth and development throughout the disease course, distinguishing PFAPA from infectious, malignant, or other systemic inflammatory diseases [9,10].

The etiopathogenesis of PFAPA remains incompletely understood. Accumulating evidence supports a central role of immune dysregulation, particularly of the innate immune system in genetically predisposed individuals. Several reports demonstrated alterations in inflammatory mediators during the febrile flares, as well as structural and functional differences in tonsillar tissue. The absence of consistent infectious triggers, the lack of sustained response to antimicrobials, and the dramatic response to corticosteroids support the autoinflammatory nature and argue against an infectious origin [11].

Genetic studies have not identified a single causative gene mutation, and PFAPA is now regarded as a complex genetic disorder. Familial clustering and genetic screening of PFAPA cohorts has identified variants linked to other inflammatory disorders like FMF and Bechet’s disease which support this concept [12,13].

The goal of PFAPA management is to reduce the frequency and severity of acute febrile episodes. Acute attacks are treated with antipyretics, nonsteroidal anti-inflammatory drugs (NSAIDs), corticosteroids, and anti-IL-1, while colchicine, cimetidine, and tonsillectomy have varying success in preventing recurrences. Additional therapeutic approaches, such as vitamin D, probiotics, and other immunomodulatory strategies, have been explored [9,14].

Although PFAPA syndrome has been addressed in several previous reviews, important advances have recently refined the understanding of its pathogenesis, genetic background, diagnostic approaches, and management. This narrative review aims to provide an updated and clinically relevant synthesis of PFAPA in children. Although PFAPA may occur in adults, the review primarily focuses on the pediatric population, with emphasis on the latest evidence on innate immune dysregulation, emerging genetic susceptibility, microbiome-related mechanisms, novel biomarkers, and evolving therapeutic strategies, including recent consensus recommendations and biologic therapies. In addition, it provides practical overviews of diagnostic criteria, laboratory investigations, differential diagnosis, and current treatment options, while highlighting current controversies, evidence gaps, and areas for future research. By combining recent scientific advances with clinically applicable guidance, this review serves as an up-to-date reference for pediatricians, rheumatologists, infectious disease specialists, and other clinicians caring for children with recurrent fevers.

## 2. Methods

This narrative review was conducted using PubMed and Scopus to identify English-language publications on PFAPA syndrome published from 1 January 2010 to 4 February 2026. The search strategy included the keywords “PFAPA,” “periodic fever, aphthous stomatitis, pharyngitis, cervical adenitis,” “Marshall syndrome,” “genetics,” “pathogenesis,” “microbiome,” “diagnosis,” “treatment,” “Behçet disease,” “familial Mediterranean fever,” and “autoinflammatory diseases.” The search strategy also combined (“PFAPA” OR “periodic fever, aphthous stomatitis, pharyngitis, and cervical adenitis” OR “Marshall syndrome”) AND (“children” OR “pediatric” OR “paediatric”). Additional targeted searches combined PFAPA with terms related to pathogenesis, genetics, diagnosis, biomarkers, treatment, and differential diagnosis, including (“PFAPA” AND (“*MEFV*” OR “*NLRP3*” OR “*TNFRSF1A*” OR “*MVK*” OR “*TNFAIP3*” OR “genetic” OR “autoinflammatory”) and (“PFAPA” AND (“familial Mediterranean fever” OR “FMF” OR “MKD” OR “TRAPS” OR “CAPS” OR “Behçet” OR “SURF” OR “cyclic neutropenia”)

The search period was selected to capture the major advances in PFAPA research, particularly in disease pathogenesis, genetic susceptibility, diagnostic approaches, and therapeutic strategies, while ensuring inclusion of the most up-to-date evidence available at the time of manuscript preparation. Earlier landmark publications published before 2010 were also included when necessary to provide historical background or to describe the original characterization of PFAPA and key diagnostic criteria.

Original research articles, review articles, clinical guidelines, consensus statements, and selected case reports providing important clinical or mechanistic insights were considered for inclusion. Titles and abstracts were screened for relevance, followed by full-text assessment of eligible publications. Reference lists of included articles were manually searched to identify additional relevant studies. Emphasis was placed on recent studies addressing disease pathogenesis, genetics, associations with FMF, Behcet’s spectrum disorders and other autoinflammatory disorders, as well as advances in diagnosis and treatment. Publications were excluded if they were unrelated to PFAPA, non-English, or lacked relevant clinical, diagnostic, pathogenic, genetic, or therapeutic information. As this is a narrative review, a formal systematic screening process and prospective recording of the number of retrieved and excluded publications were not performed and no formal systematic review protocol or risk-of-bias assessment was performed.

## 3. Pathogenesis

The pathogenesis of PFAPA remains incompletely elucidated; however, current evidence suggests a multifactorial process predominantly driven by genetic susceptibility, immune dysregulation, and aberrant inflammatory responses within tonsillar tissue. Furthermore, other influences like microbiomes as well as vitamin D deficiency have also been proposed as potential modulators of the disease expression and flare frequency [15,16,17].

### 3.1. Genetics

Genetic evaluation of children with PFAPA does not always identify a single pathogenic variant that fully explains the clinical phenotype. Some patients carry variants of uncertain significance (VUS), either alone or in combination with pathogenic or likely pathogenic variants. However, the clinical significance of these findings in PFAPA remains uncertain, and current evidence is insufficient to establish a causal role for these variants or to confirm PFAPA as part of a broader genetic autoinflammatory continuum. PFAPA-like manifestations including periodic fever, aphthous stomatitis, pharyngitis, and lymphadenitis are often observed alongside genetic alterations involving different innate immune pathways. This pattern supports the concept that PFAPA may arise from polygenic or multifactorial immune dysregulation and may clinically overlap with other autoinflammatory conditions, including FMF and Behçet-like syndromes. Approximately one-third of patients evaluated for suspected autoinflammatory disease harbor variants in more than one autoinflammatory gene, with diverse combinations of pathogenic, likely pathogenic, and VUS influencing clinical presentation. These complex genetic profiles may modify disease severity, contribute to heterogeneous or overlapping phenotypes, and account for cases classified as mixed autoinflammatory disorders (MAIDs), even in the absence of clearly causative mutations. However, the presence of a VUS does not establish causality or confirm the diagnosis of PFAPA, and its clinical significance should be interpreted cautiously in the context of the patient’s phenotype and available genetic evidence. Whether such variants contribute to disease susceptibility or modify the clinical phenotype remains uncertain and requires further investigation. Careful longitudinal monitoring of these patients is therefore crucial to better define disease trajectories, enhance diagnostic classification, and inform long-term prognostic considerations [13].

#### 3.1.1. PFAPA and FMF

Berkun et al., examined the influence of *MEFV* gene variants on the clinical presentation of PFAPA in 124 pediatric patients, found that nearly half of the cohort harbored an *MEFV* variant, most frequently M694V; however, none met FMF diagnostic criteria, and colchicine was not effective in preventing attacks. Children with *MEFV* variants showed shorter and less regular periodic attacks, fewer aphthous lesions, and lower corticosteroid doses to control the febrile episodes. Aside from these differences, clinical manifestations, laboratory parameters, and long-term outcomes were similar between *MEFV* carriers and non-carriers. Overall, these findings suggest that *MEFV* functions as a disease-modifying gene in PFAPA, leading to a milder phenotype rather than reflecting occult FMF [18].

Moreover, Serum amyloid A (SAA), which is widely tested in FMF as a marker of flare and in between the attacks as an indicator for amyloidosis, is recently found to be a highly sensitive and specific indicator of PFAPA flares and demonstrates superior diagnostic performance compared with CRP and ESR. These observations support the laboratory overlap between FMF and PFAPA [19].

Another Turkish study by Konte et al., who evaluated 131 children who fulfilled diagnostic criteria for both PFAPA and FMF, highlighted a clinical overlap within autoinflammatory diseases. Most patients carried *MEFV* gene variants, particularly exon 10 mutations, and exhibited combined PFAPA features (pharyngitis, aphthous stomatitis) and FMF-like serositis symptoms. Colchicine was effective in approximately two-thirds of patients with *MEFV* mutations predicting a favorable response, whereas PFAPA family history, aphthous stomatitis, and tonsillopharyngitis were associated with colchicine resistance. Tonsillectomy led to remission in most colchicine-resistant patients, including those who met the FMF diagnostic criteria. These findings support the concept of a “gray zone” between PFAPA and FMF, emphasizing the need to individualize treatment strategies and reconsider rigid diagnostic boundaries in periodic fever syndromes [20].

#### 3.1.2. PFAPA in the Context of Behcet’s Spectrum Disorders (BSDs)

PFAPA, Behçet syndrome, and recurrent aphthous stomatitis (RAS) share genetic and pathogenic features and have been termed “Behçet spectrum disorders”, with PFAPA representing an intermediate phenotype between the mild RAS and the more severe Behçet disease. In context of complex genetic disorders like PFAPA, common variant mutations may have a role in pathogenesis. Familial clustering and recurrent aphthous ulcers have been reported with greater frequencies among first-degree relatives of children with PFAPA than controls. Class I HLA alleles have been implicated as major genetic susceptibility factors for Behçet’s disease, with HLA-B*51:01 consistently identified as the strongest associated risk allele. Although PFAPA is associated with several class I HLA alleles, these genetic associations are largely distinct from those seen in Behçet disease and RAS. Genome-wide association studies further expanded this genetic landscape by identifying multiple non-HLA risk loci. Many of these variants are relatively common in the general population but show altered frequencies in affected individuals and cluster near genes regulating immune and inflammatory pathways, such as *IL10*, *IL23R–IL12RB2*, *STAT4*, *IL12A*, *CCR1–3*, *TLR4*, and *TNFAIP3*, influencing their gene expression. In addition, rare coding variants with low minor allele frequencies have been described in genes including *MEFV*, *TLR4*, and *IL23R* [12,21]. Collectively, some of these genetic variants contribute to enhanced activation of Th1 and Th17 immune pathways. Notably, increased Th1 activity has been demonstrated in both tonsillar tissue and peripheral blood of children with PFAPA, particularly during febrile flares [22].

Clinically, some patients exhibit overlapping features of PFAPA and Behçet’s disease. These include individuals initially diagnosed with PFAPA who subsequently develop Behçet’s disease manifestations such as genital ulcers, as well as adults with Behçet’s disease who experienced PFAPA-like periodic fevers in childhood [12].

#### 3.1.3. Potential Role of Rare VUS

Kolly and colleagues evaluated PFAPA patients for three *NLRP3* variants (V198M, Q703K, and R488K) and found these variants in 20% of cases, exceeding the expected population frequency of approximately 12%, with the R488K variant being significantly overrepresented. Moreover, monocyte-derived IL-1β production was shown to be dysregulated, supporting the concept that alterations in inflammasome-related genes may contribute to PFAPA susceptibility within a polygenic or multifactorial framework [23].

*CARD8*, a proposed negative regulator of *NLRP3* inflammasome activity, has been implicated in PFAPA patients. Heterozygous *CARD8-FS* were identified in 14% of PFAPA patients, a rate significantly exceeding that observed in ethnically matched controls with higher frequency of oral aphthae and increased inflammatory symptoms between PFAPA episodes among carriers [24].

The low-penetrance *TNFRSF1A* variant R92Q has been identified in children with periodic fever syndromes, including PFAPA. Unlike pathogenic *TNFRSF1A* variants causing TNF-receptor-associated periodic syndrome (TRAPS), R92Q has minimal impact on TNFR1 structure and function and is associated with a milder clinical course, higher rates of spontaneous remission, and a phenotype that more closely resembles PFAPA than TRAPS. Long-term follow-up suggests that R92Q acts as a genetic modifier, influencing inflammatory responses and producing an intermediate phenotype rather than serving as a direct cause of PFAPA [25]. In contrast, based on low prevalence, other reports concluded that *TNFRSF1A* mutations are unlikely to play a significant role in the pathogenesis of PFAPA [15].

SPAG7, a gene involved in antiviral and inflammatory pathways, has been proposed by Bens et al. as a potential PFAPA susceptibility gene. The authors suggested that somatic variants or epigenetic alterations of SPAG7 might contribute to disease mechanisms. However, as a SPAG7 genetic variant was identified in only a single patient, its relevance to PFAPA remains uncertain and requires further validation [26].

ALPK1 plays a role in innate immune signaling by activating its kinase function, ultimately leading to stimulation of the *NLRP3* inflammasome with release of multiple cytokines. In 2019, a multigenerational family with several PFAPA-affected members was evaluated for variants in autoinflammatory pathway genes. PFAPA segregated across two generations in a pattern suggestive of autosomal dominant inheritance, and a single ALPK1 variant was found to be consistently shared among affected relatives [27].

Multiple additional variants have been evaluated, including several NLRP polymorphisms (Q703K, S726G, P340P, and P200T), comprehensive analysis of inflammasome-related genes such as the full coding and promoter regions of AIM2 and the *NLRP3* gene, as well as the *MVK* gene implicated in mevalonate kinase deficiency/hyperimmunoglobulin D syndrome (MKD/HIDS). However, none of these variants have exhibited a meaningful association with PFAPA [23,28].

The lack of a single gene harboring mutations in protein-coding regions across all affected individuals suggests that PFAPA does not arise from a single genetic defect but rather reflects substantial genetic heterogeneity. This supports models of Mendelian inheritance with variable genetic causes or, alternatively, an oligogenic or complex mode of transmission. Undetected variants in noncoding or insufficiently covered genomic regions may also contribute and warrant further investigation. The presence of multiple inflammasome-related variants in some patients is compatible with an oligogenic model; however, because such variants are also found in unaffected populations, their pathogenic relevance remains uncertain and should be interpreted cautiously. In clinical practice, genetic testing may nonetheless be appropriate in atypical PFAPA cases or when alternative autoinflammatory conditions, such as FMF or MKD, are suspected [29,30].

### 3.2. Immune Dysregulation

Accumulating evidence indicates that PFAPA is primarily driven by immune dysregulation in genetically predisposed individuals, involving both innate and adaptive immune pathways. During disease flares, there is marked activation of inflammasome-related mechanisms, with increased production of pro-inflammatory cytokines such as interleukin-1β (IL-1β) and interleukin-18 (IL-18). This inflammatory response is accompanied by elevated levels of tumor necrosis factor (TNF), interleukin-6 (IL-6), C-reactive protein (CRP), and SAA. Enhanced activation of monocytes and neutrophils has also been observed, along with increased expression of inflammatory markers including S100A12 and MRP8/14. In addition, IL-1β contributes to dysregulated T-cell activation, which promotes the release of interferon- gamma (IFN-γ), which suppresses the production of the anti-inflammatory interleukins (IL-4 and IL-10). In parallel, there is an upregulation of T-cell associated chemokines, including CXCL9 and CXCL10 [9,15,31].

Several reports demonstrated high rates of clinical remission following tonsillectomy, with histological features suggestive of chronic tonsillar inflammation. Subsequent studies identified altered T-cell populations within PFAPA tonsils, with greater CD8+ T cells in PFAPA tonsils supporting a potential role for T-cell mediated immune mechanisms. However, conflicting observations reported reduced CD8^+^ T-cell density in germinal centers compared with controls. More recent analyses described structural changes such as enlarged germinal centers and epithelial alterations, further indicating immune dysregulation within tonsillar tissue [22,32,33].

Beyond structural and cellular abnormalities, emerging evidence implicates metabolic dysregulation in PFAPA pathogenesis. During inflammatory flares, immune cells undergo metabolic reprogramming characterized by a shift toward glycolysis to sustain inflammatory activity, while oxidative phosphorylation predominates during resolution. Disruption of mitochondrial ATP generation or mTOR signaling was shown to impair neutrophil chemotactic function. Inhibition of mitochondrial pyruvate oxidation promotes a proinflammatory state by favoring glycolytic metabolism, enhancing *NLRP3* inflammasome activation and IL-1β production [34]. The identification of immunometabolism as a pivotal modulator of inflammatory responses has expanded therapeutic horizons, suggesting that modification of metabolic pathways in immune and vascular cells may offer effective means to control inflammation. Consequently, deeper characterization of metabolic programs within inflammatory cells may enable the development of targeted interventions to limit pathological inflammatory activity.

### 3.3. Vitamin D Insufficiency/Deficiency

Approximately one third of children with PFAPA have reduced serum 25-hydroxyvitamin D levels, although single measurements may not accurately reflect long-term vitamin D status. Beyond its classical role in bone metabolism, vitamin D exerts pleiotropic effects, including immunomodulatory ones. It influences T-cell proliferation, dendritic cell activity, and cytokine production, thereby contributing to immune homeostasis. Vitamin D deficiency may arise from intrinsic factors, such as genetic variations affecting vitamin D metabolism, bioavailability, or receptor function, as well as extrinsic factors including limited sunlight exposure, inadequate dietary intake, malabsorption syndromes, hepatobiliary or pancreatic disorders, and medications that can affect vitamin D metabolism. Multiple clinical studies have demonstrated an association between low vitamin D levels and PFAPA. Moreover, reduced vitamin D levels have been correlated with inflammatory markers as CRP and proposed as a potential risk factor for recurrent disease flares. Importantly, vitamin D supplementation may reduce the frequency and duration of PFAPA episodes, supporting the modulatory role of vitamin D in PFAPA activity. Nevertheless, whether vitamin D deficiency contributes to PFAPA pathogenesis or represents a consequence of recurrent inflammation is not fully established. Further studies are needed to clarify the potential role of vitamin D in PFAPA [35,36].

### 3.4. Microbiome Dysbiosis

Numerous environmental factors have been explored in relation to PFAPA syndrome; however, no specific trigger has been definitively identified. Increasing attention has focused on the oral cavity, which harbors a highly diverse microbiota distributed across multiple anatomical niches, including the tonsils. Although alterations in microbial composition have been linked to recurrent upper airway infections in children, the precise relationship between oral or tonsillar microbiota and PFAPA flares remains unclear, and an infectious etiology has not been established. Histopathological analyses of tonsillar tissue from PFAPA patients commonly demonstrate chronic inflammation with lymphoid hyperplasia and heterogeneous immune patterns. Microbiome studies using next-generation sequencing have identified differences in tonsillar bacterial communities in PFAPA compared with controls, including reduced representation of Streptococcus species and increased abundance of cyanobacteria. These findings have led to the hypothesis that impaired regulation of innate immune responses may result in inappropriate inflammatory reactions to otherwise commensal oral microorganisms, potentially explaining the periodic nature of PFAPA episodes [16,37,38].

Based on this concept, modulation of the oral microbiome has emerged as a potential therapeutic strategy. Probiotic supplementation with Streptococcus salivarius K12, an oral commensal strain shown to reduce respiratory infections in children, has been associated with clinical improvement in PFAPA as it may help reestablish the ability of innate immune cells to appropriately identify oral microbiota as harmless commensals. Prolonged administration has been reported to decrease the frequency and duration of PFAPA febrile episodes and reduce corticosteroid use. However, these findings remain preliminary, and whether microbiome alterations contribute to PFAPA pathogenesis or result from the disease or its treatment remains unclear [39].

## 4. Clinical Manifestations

Stereotypical, recurrent febrile episodes are characteristic of PFAPA, although the frequency and regularity of episodes may vary among patients. Episodes typically last 3–7 days (most commonly 4–5 days) and recur at intervals of approximately 2–8 weeks, with most occurring every 3–6 weeks. These episodes associated with pharyngitis, aphthous ulcers and cervical lymphadenopathy are considered the cardinal clinical manifestations of PFAPA. Children are asymptomatic between the episodes. Nonetheless, oral aphthous lesions and malaise can sometimes be present even between the episodes. Fever typically has a sudden onset and may be associated with chills and is generally resistant to antimicrobials and antipyretics. Nonspecific prodromal symptoms, including malaise, irritability or behavioral changes, and sore throat, can precede the febrile episode. Fever commonly rises to 39–40.5 °C and then resolves abruptly to baseline. Pharyngitis, reported in more than 90% of patients, may present as erythematous or exudative. Tonsils may appear normal or enlarged, and in very young children pharyngeal involvement may be suggested only by excessive drooling or decreased oral intake. Cervical lymphadenitis is observed in approximately 75% of cases. It usually involves the anterior cervical lymph nodes, usually bilateral, and mildly to moderately tender without associated overlying skin abnormalities. Aphthous stomatitis occurs in roughly 50% of patients and typically involves the inner lips or buccal mucosa, with occasional lesions affecting the posterior pharynx. The ulcers are usually small, approximately 1 cm in diameter, and may be overlooked without careful examination. Compared with Behçet disease, PFAPA aphthous ulcers are smaller, and less painful. In addition to these classic manifestations, the episodes may be associated with non-specific symptoms such as abdominal pain, vomiting, diarrhea, headache, arthralgia, myalgia, and fatigue [8,9,10,40].

Some patients with recurrent febrile episodes may not exhibit the full spectrum of classic PFAPA manifestations, such as pharyngitis, cervical adenitis, or aphthous stomatitis. Although rapid clinical improvement following systemic glucocorticoids and favorable outcomes after tonsillectomy have been reported in some patients with incomplete PFAPA features, neither treatment response is specific enough to establish the diagnosis. Such presentations should therefore be interpreted cautiously, as PFAPA may encompass a wider phenotypic spectrum but with careful consideration of alternative causes of recurrent fever [41].

Although PFAPA typically begins in early childhood and often resolves spontaneously around puberty, persistent disease, recurrence after remission and adult-onset cases have been reported. Compared with children, adults are less likely to exhibit strict periodicity of febrile episodes or prominent pharyngitis, but more frequently report systemic manifestations such as chest pain, headache, arthralgia, myalgia, ocular symptoms, and rash [42].

## 5. Laboratory Investigations

No specific diagnostic laboratory test is available for PFAPA so far. During attacks, patients may exhibit neutrophilia, lymphopenia, eosinopenia, and monocytosis, along with elevated inflammatory markers including CRP, ESR, serum amyloid A (SAA), S100 calcium-binding protein A8/A9 (S100A8/A9), also known as calprotectin and S100 calcium-binding protein A12 (S100A12). These findings, however, lack specificity. In contrast, procalcitonin levels remain normal during PFAPA flares but, typically rise in bacterial infections aiding in differential diagnosis. In between the attacks, inflammatory markers and CBC changes return to normal which favors PFAPA diagnosis. However, none of these biomarkers can be considered specific for PFAPA or can independently confirm the diagnosis. Their clinical utility lies primarily in supporting the assessment of inflammation and helping distinguish PFAPA from infectious and other inflammatory conditions, when interpreted in conjunction with the clinical presentation [9,23,40].

Routine genetic testing is not indicated. Nevertheless, it may be valuable in selected patients with atypical features, incomplete clinical criteria, early or severe disease, or poor therapeutic response. In such cases, testing helps exclude monogenic autoinflammatory disorders with overlapping phenotypes, including FMF, MKD, and TRAPS, so it serves primarily as a differential diagnostic rather than a confirmatory test for PFAPA. Broad genetic panels should be interpreted cautiously because they may identify variants of uncertain significance (VUS) that are difficult to interpret and do not establish a diagnosis [43].

## 6. Diagnosis

PFAPA is a clinical diagnosis of exclusion established after ruling out other causes of recurrent fever, including repeated infections, cyclic neutropenia (cyclic hematopoiesis), malignancy-associated fever, early onset inflammatory bowel disease, and monogenic autoinflammatory syndromes. Accurate diagnosis relies on careful assessment of fever periodicity and detailed physical examination during attacks, with fever diaries serving as a useful tool to define symptom patterns, supported by high inflammatory markers during the attack and the return to normal in between. Establishing diagnosis helps reduce caregiver anxiety, limits unnecessary investigations, and avoids inappropriate treatments such as unwarranted antibiotics [11,44].

Since its initial description by Marshall et al. in 1987, several refinements of diagnostic and classification criteria were published as illustrated in Table 1 [3,4,5,6,7].

In addition to established diagnostic/classification criteria, although it is not specific, a dramatic response of fever and possibly pharyngitis, after one or two doses of corticosteroids may help in distinguishing PFAPA from infectious etiologies and hereditary autoinflammatory disorders [9].

## 7. Differential Diagnosis

### 7.1. Monogenic Autoinflammatory Disorders

A major diagnostic challenge in PFAPA lies in distinguishing it from other periodic fever syndromes and other hereditary autoinflammatory disorders, especially FMF, MKD, TRAPS, cryopyrin-associated periodic syndromes (CAPS, recently named as *NLRP3*-associated autoinflammatory disease), and haploinsufficiency of A20 (HA20). Although recurrent fever is a common feature across most of these disorders, differences in attack duration, associated clinical manifestations, and underlying genetic profiles provide important clues for accurate differential diagnosis.

Compared with PFAPA, febrile episodes in FMF are typically shorter, lasting 1–3 days, and lack the strict periodicity of PFAPA. The interval between FMF attacks is variable, even within the same patient, and episodes may be precipitated by triggers such as physical or emotional stress. Unlike PFAPA, inflammatory markers in FMF often fail to normalize completely between attacks in some patients, reflecting ongoing subclinical inflammation which can be complicated by amyloidosis, which is an important but uncommon complication, occurring mainly in patients with sustained, poorly controlled inflammation and persistently elevated SAA levels. In addition, FMF flares are not fully suppressed by corticosteroid therapy. Identification of pathogenic *MEFV* gene variants, particularly in homozygous or compound heterozygous states, further supports the diagnosis of FMF. Furthermore, both PFAPA and FMF may co-occur together particularly in Mediterranean regions [7,40,45,46,47].

Although febrile episodes in MKD often last 3–7 days, like PFAPA, the episodes of MKD are typically more variable in duration and timing and lack the strict periodicity of PFAPA. MKD flares are frequently precipitated by vaccinations and are commonly associated with obvious systemic manifestations, including diarrhea, vomiting, abdominal pain, rash, lymphadenopathy, and arthralgia. Interestingly, immunoglobulin D levels may be elevated in both MKD and PFAPA. Definitive diagnosis of MKD depends on the identification of pathogenic variants in the *MVK* gene. Moreover, increased urinary excretion of mevalonic acid could support the diagnosis. In contrast to PFAPA, MKD episodes generally demonstrate a partial response to corticosteroid therapy [7,48].

In contrast to PFAPA, TRAPS is characterized by more prolonged febrile episodes, typically lasting longer than 7 days up to several weeks. TRAPS lacks the strict periodicity of PFAPA and is commonly associated with distinctive clinical features, including periorbital edema and migratory rash. A positive family history is frequently present, reflecting the autosomal dominant pattern of TRAPS, confirmed by identification of pathogenic variants in the *TNFRSF1A* gene. Additionally, febrile episodes in TRAPS do not respond reliably to single-dose corticosteroid therapy [7].

*NLRP3*-associated autoinflammatory diseases (formerly termed as CAPS) are characterized by a more continuous or chronic inflammatory course rather than discrete, self-limited regular febrile episodes of PFAPA. Fever in CAPS is often accompanied by urticarial-like rash, along with systemic manifestations such as sensorineural hearing loss and ocular involvement. Neurologic features may also be present in more severe phenotypes. Diagnosis is confirmed by identification of pathogenic variants in the *NLRP3* gene. Unlike PFAPA, inflammatory activity in CAPS does not resolve completely between episodes and does not show a dramatic response to single dose of corticosteroids [7].

HA20 can clinically overlap with PFAPA as both exhibit recurrent fever and oral ulcers but fever episodes in HA20 lack the strict periodicity of PFAPA and are frequently accompanied by prominent mucocutaneous and gastrointestinal manifestations, including recurrent oral, genital, and gastrointestinal ulcers. Systemic features such as arthritis or arthralgia, rashes, uveitis, neurologic involvement, and autoimmune thyroid disorders. Also, gastrointestinal symptoms such as abdominal pain, diarrhea, vomiting, or constipation are particularly frequent in HA20. Additionally, HA20 is confirmed by identifying pathogenic variants in *TNFAIP3* [49].

### 7.2. Syndrome of Undifferentiated Recurrent Fever (SURF)

SURF represents a heterogeneous group of autoinflammatory disorders characterized by recurrent febrile episodes with systemic inflammation not fulfilling diagnostic criteria for PFAPA or any defined monogenic periodic fever syndrome. The underlying pathophysiology of SURF remains poorly defined, with genetic, environmental, and epigenetic factors thought to contribute to disease expression. Clinically, SURF patients frequently display overlapping features of multiple autoinflammatory syndromes, with more prominent systemic manifestations such as abdominal pain, arthralgia, myalgia, and rash. Genetic testing in SURF patients often reveals variants of uncertain significance (VUR) rather than pathogenic mutations. Compared with PFAPA, SURF tends to persist beyond early childhood, shows limited response to corticosteroids and tonsillectomy, and is more likely to respond to colchicine therapy and anti-interleukin (IL)-1 [50,51,52].

### 7.3. Behçet Disease

Both PFAPA and Behçet disease are characterized by recurrent febrile episodes accompanied by oral ulceration. However, PFAPA typically begins in early childhood and is dominated by pharyngotonsillitis and cervical lymphadenopathy, whereas Behçet disease usually presents in older children and adolescents with prominent mucocutaneous involvement, including oral and genital ulcers, and may feature gastrointestinal, neurologic, vascular, and ocular manifestations [53].

### 7.4. Cyclic Neutropenia (Cyclic Hematopoiesis)

Cyclic neutropenia is a rare autosomal dominant disorder and represents the only childhood condition with truly periodic fever that closely mimics PFAPA. Febrile episodes typically recur at fixed intervals of approximately 21 days. The key distinguishing feature is profound neutropenia during attacks, with absolute neutrophil counts falling below 500/mm^3^, which is not observed in PFAPA. Mucosal involvement is usually more severe, with extensive aphthous stomatitis and prominent gingivitis, exceeding the oral manifestations seen in PFAPA. Unlike PFAPA, episodes do not respond to single-dose corticosteroids. Diagnosis requires serial complete blood counts demonstrating cyclical neutropenia and can be genetically confirmed by identifying pathogenic variants in the *ELANE* gene [8,40].

### 7.5. Infections

Recurrent viral or bacterial infections in childhood usually occur irregularly, often seasonally, and lack the predictable periodicity typical of PFAPA. The diagnosis of PFAPA is supported by repeatedly negative cultures, absence of sick contacts and respiratory symptoms such as cough or rhinorrhea, poor response to antibiotics, and rapid resolution of fever after a single corticosteroid dose. PFAPA is frequently mistaken for recurrent streptococcal pharyngitis; however, streptococcal disease is characterized by positive Group A Streptococcus cultures and antibiotic responsiveness, whereas PFAPA episodes are mostly microbiologically negative, stereotyped, and corticosteroid responsive [40] (Table 2).

**Table 2:** PFAPA differential diagnosis.

Condition	Fever Pattern	Key Distinguishing Features	Diagnostic Clues
PFAPA	Periodic, 3–7 days	Aphthous stomatitis, pharyngitis, cervical adenitis	Clinical diagnosis; inflammatory markers often normalize between attacks, rapid response to corticosteroids
FMF	1–3 days (lacks the strict periodicity)	abdominal/chest pain, arthritis, erysipelas-like rash, positive family history	*MEFV* variants, inflammatory markers often fail to normalize completely between attacks
MKD	3–7 days (lacks the strict periodicity)	Diarrhea, vomiting, abdominal pain, rash, lymphadenopathy,	*MVK* variants; urinary mevalonic acid
TRAPS	longer than 7 days up to several weeks	Migratory rash, severe myalgia, periorbital edema,	*TNFRSF1A* variants
CAPS	Persistent/chronic	Urticarial-like rash, neurologic symptoms, hearing loss	*NLRP3* variants
HA20	Irregular/recurrent	Oral/genital/GI ulcers, arthritis, rash, uveitis	*TNFAIP3* variants
SURF	Variable/recurrent	Abdominal pain, myalgia, fatigue; atypical PFAPA features	Diagnosis of exclusion; inconclusive genetic testing
Behçet disease	Recurrent/variable	Oral and genital ulcers, uveitis, skin/neurological manifestations	Clinical diagnosis
Cyclic neutropenia	~21-day cycles	Severe neutropenia, deep oral ulcers, recurrent bacterial infections	Serial CBC; *ELANE* variants
Infections	Sporadic	Exudative tonsillitis, sick contacts, focal infectious features	Positive culture/PCR or other pathogen testing

## 8. Treatment

The management of PFAPA focuses on suppressing inflammation by rapidly controlling acute febrile episodes and decreasing the frequency or intensity of recurrences to improve quality of life. Systemic corticosteroids are the most effective pharmacologic therapy for aborting acute attacks, whereas tonsillectomy represents the most effective intervention for long-term reduction or prevention of recurrent episodes. Choice of treatment modality is shared between the patient/parents and the physician [40].

### 8.1. Treatment of Acute Attacks

#### 8.1.1. Antipyretics and Nonsteroidal Anti-Inflammatory Drugs (NSAIDs)

Although initially used, antipyretics as acetaminophen or the more effective NSAIDs as ibuprofen can provide adequate symptom relief, suitable for patients with mild PFAPA and for families who want to avoid corticosteroids, long-term medications, or surgical intervention by tonsillectomy [9].

#### 8.1.2. Corticosteroids

Corticosteroids are the most effective treatment in aborting acute attacks of PFAPA with dramatic resolution of fever within hours and other symptoms within a relatively longer time in about 90% of PFAPA pediatric patients and in about 85% of adults [54,55].

Variable dose regimens have been prescribed ranging from 0.5 mg/kg to 2 mg/kg. A randomized controlled trial by Yazgan et al., including 86 PFAPA patients, found that low-dose prednisolone (0.5 mg/kg) was as effective as higher doses (2 mg/kg) [56].

The CARRA PFAPA Working consensus reported use of prednisolone of 1 mg/kg by 64% of physicians or 2 mg/kg by 29 of physicians. A single dose of prednisolone is given at fever onset. It is well established that although corticosteroids rapidly terminate febrile episodes in PFAPA, they do not prevent future attacks and may even shorten the interval between flares in approximately 25–50% of patients. In addition, although intermittent single-dose corticosteroid therapy is generally regarded as safe, transient adverse effects such as irritability, behavioral changes, and sleep disturbance have been reported. Persistence of short attack intervals (≤2 weeks) despite this adjustment prompts transition to an alternative therapeutic strategy like colchicine/cimetidine or tonsillectomy rather than continued corticosteroid escalation [9,14].

#### 8.1.3. IL-1 Inhibitors

Short-acting anti IL-1 with anakinra has been shown to rapidly abort febrile episodes with normalization of inflammatory markers when administered during attacks, although relapse within days has been observed in some patients. Similarly, an adult PFAPA patient with disease refractory to standard therapies, including corticosteroids, colchicine, and tonsillectomy, achieved complete resolution of febrile attacks following treatment with anakinra and later with canakinumab after secondary loss of response after 24 months. Despite the central role of IL-1 signaling in PFAPA pathophysiology, sustained prevention of attacks with long-acting IL-1 inhibitors appears inconsistent, highlighting differences between PFAPA and other IL-1 mediated disorders such as FMF. Given the usually benign and self-resolving nature of PFAPA in childhood, IL-1 inhibitors should be considered only in highly selected, treatment-refractory cases after careful assessment of benefit, cost, and long-term risk. Given the limited pediatric evidence and lack of randomized controlled trials, the role of anti-IL-1 therapies in PFAPA remains uncertain, and no clear consensus has been established regarding their routine use [57,58].

### 8.2. Preventive or Prophylactic Treatment

#### 8.2.1. Colchicine

Colchicine exerts anti-inflammatory effects via binding to tubulin and inhibiting microtubule polymerization, thereby interfering with multiple cellular processes such as cytokine release, cell migration, and immune activation. Although its precise mechanism in PFAPA remains unclear, evidence of inflammasome activation and increased IL-1β during attacks supports an autoinflammatory basis, analogous in part to FMF, where Th1-mediated responses are sensitive to IL-1 modulation. In PFAPA, colchicine is primarily used as a prophylactic agent rather than for aborting acute flares. In a large study, prophylactic colchicine therapy was associated with a lengthening of the interval between attacks in 85% of patients receiving regular treatment (n = 303), with mean interval between episodes increased significantly from 18.8 ± 7.9 days to 49.5 ± 17.6 days, supporting the colchicine efficacy [59].

In another cohort, colchicine was administered to 122 PFAPA patients. Following treatment with colchicine, complete or near-complete remission of attacks was achieved in 46.7%, while a partial clinical response was observed in 48.4%; only 6 patients (4.9%) showed no appreciable benefit. Colchicine therapy was additionally associated with a marked reduction in attack duration, with the median length decreasing from 4 days (IQR 4–5) to 2 days (IQR 1–2.5). In addition, attack frequency was significantly reduced, with episodes occurring approximately every 4 weeks (IQR 3–4) before treatment compared with every 10 weeks (IQR 8–24) after initiation of colchicine [60].

Several studies examining the impact of *MEFV* variants on colchicine responsiveness revealed inconsistent results. Gunes et al. reported that colchicine Prophylactic therapy resulted in a significantly greater reduction in attack frequency among patients carrying an *MEFV* gene variant (96%, n = 54) compared with those without the variant (80%, n = 122), (*p* = 0.003) [59]. In contrast to this, Recent data indicate no overall difference in treatment response between patients with and without *MEFV* variants; however, individuals carrying the M694V variant show a smaller improvement in disease activity scores after colchicine initiation. The presence of *MEFV* variants does not appear to affect the interval between starting therapy and the first subsequent attack. From a clinical perspective, lack of clear benefit after one month should prompt reassessment of adherence, dosing, or alternative therapies, while patients with the M694V variant may require a longer treatment duration to accurately evaluate colchicine efficacy [60,61].

There is no consensus regarding the optimal duration of colchicine therapy in patients with PFAPA so far. In a preliminary study, Sönmez et al. proposed that colchicine could be stopped in patients who are heterozygous for *MEFV* variants after a sustained symptom-free period of two years. Following treatment discontinuation, clinical follow-up is advised to monitor for recurrence of attacks and changes in acute phase inflammatory markers [62]. Given the typical self-limiting nature of PFAPA, a shorter course of colchicine may be sufficient in patients who are heterozygous for *MEFV* variants. Nevertheless, additional studies are needed to establish the optimal duration of therapy in PFAPA patients.

Colchicine is generally well tolerated at daily doses of 0.5–1 mg, with gastrointestinal symptoms being the most frequently reported adverse effects, occurring in roughly 10% of patients. These reactions may be partly related to the induction or exacerbation of lactose intolerance, although additional mechanisms are likely involved. Overall, colchicine could be an effective prophylactic therapy in PFAPA, particularly in patients in whom corticosteroid use shortens the interval between febrile episodes [63].

#### 8.2.2. Cimetidine

Cimetidine, an H2-receptor antagonist, possesses immunomodulatory effects, including suppression of chemotaxis and T-cell activation [63]. Approximately 43% of PFAPA patients experienced clinical benefit with cimetidine prophylaxis [4].

According to the CARRA consensus plans, cimetidine is included as a prophylactic option for children with PFAPA who require preventive therapy, typically administered at approximately 20–40 mg/kg/day divided into two doses. Its use is supported by limited evidence suggesting modest efficacy in reducing episode frequency, and it is generally considered in patients who have frequent flares or shortened inter-episode intervals despite on-demand corticosteroid therapy [14].

Raeeskarami et al. conducted a randomized controlled trial (RCT) comparing cimetidine and colchicine in 67 pediatric patients with PFAPA. Participants were assigned to receive either prednisolone plus colchicine (n = 36) or prednisolone plus cimetidine (n = 31). Both treatment strategies led to a significant reduction in the frequency of febrile episodes after intervention, with no statistically significant difference in efficacy between both groups (*p* = 0.88). Furthermore, among 44 patients tested for *MEFV* mutations, treatment response to colchicine did not differ between *MEFV* positive and negative patients [64].

#### 8.2.3. Tonsillectomy/Adenotonsillectomy

Tonsillectomy, performed either alone or with adenoidectomy, is considered one of the most effective long-term treatment options for PFAPA by achieving complete remission or marked decrease in episode frequency. Its benefit is presumed to result from eliminating the source of persistent immune stimulation, thereby attenuating systemic inflammatory responses and improving clinical manifestations [9].

In a prospective RCT, 26 children with at least five PFAPA episodes were assigned either to tonsillectomy or observation. Participants were monitored for one year with the option of surgery for controls after six months if attacks persisted. At six months, all children in the surgical group were symptom-free compared with half of those managed conservatively, a statistically significant difference (*p* < 0.001). Most symptomatic controls subsequently underwent tonsillectomy, while one child continued to have milder recurrent episodes without surgery indicating that tonsillectomy is an effective therapeutic option for PFAPA, although spontaneous improvement may occur in a subset of patients [65].

In another RCT including 39 children with PFAPA, participants were allocated to adenotonsillectomy (n = 19) or conservative management (n = 20) and were followed prospectively for 18 months. Complete remission occurred in 63% of surgically treated patients compared with 5% of controls, demonstrating a statistically significant benefit. Moreover, the mean number of attacks during follow-up was markedly lower in the surgical group (0.7 ± 1.2) than the control group (8.1 ± 3.9), and residual episodes were milder in severity supporting adenotonsillectomy as an effective therapeutic option for children with PFAPA [66].

In a recent multicenter RCT, 16 children with PFAPA were assigned either to tonsillotomy (partial tonsillectomy) or to observation alone. At follow-up, 87.5% of patients in the tonsillotomy group were symptom-free compared with 25% in the control group, and the number of PFAPA-related fever days was significantly lower in the surgical arm (2.6 SD 3.7) vs. (8.0 SD 6.5) in controls. All children in the observation group ultimately required rescue tonsillectomy, whereas none in the tonsillotomy group did. Despite limited tissue removal and no adenoidectomy, the procedure appeared beneficial. The short-term follow-up period limits conclusions regarding long-term efficacy, and further studies are warranted [67].

A recent retrospective study assessing long-term outcomes after tonsillectomy in PFAPA patients found that, over a median follow-up of eight years, 63% achieved complete remission, 17% experienced prolonged intervals between episodes, while 20% continued to report non-febrile PFAPA-related manifestations, most commonly recurrent aphthous ulcers [68].

Manthiram et al. recently reported that complete remission following tonsillectomy was more likely in patients whose febrile episodes were promptly aborted by glucocorticoids, those exhibited exudative pharyngitis, and those lacking rash or arthralgia/myalgia during preoperative flares. Among patients with ongoing episodes after surgery, clinical improvement had been achieved with prophylactic therapy using cimetidine.

Recent systematic reviews and meta-analyses support a potential benefit of surgical treatment in PFAPA. Cavalcante et al. reported a 72% reduction in persistent symptoms following tonsillectomy or tonsillotomy compared with non-surgical management (RR: 0.28, 95% CI: 0.12–0.68; I^2^ = 22%; *p* = 0.005); however, the analysis included only three randomized controlled trials, warranting cautious interpretation and further confirmation in larger studies [69].

Noy et al. recently, conducted a systematic review and network meta-analysis of nine studies involving 691 patients and found that surgical treatment was associated with a higher likelihood of symptom resolution than medical treatment (OR: 11.7, 95% CI: 2.14–63.94), although substantial heterogeneity was observed. In analyses restricted to randomized controlled trials, both tonsillectomy and intracapsular tonsillectomy were associated with greater symptom resolution. The pooled complication rate was 6.6%, while symptom recurrence occurred in 13.6% after tonsillectomy and 37.5% after intracapsular tonsillectomy. Despite these findings, the limited evidence base and heterogeneity across studies warrant cautious interpretation and further randomized studies [70].

Only limited evidence exists regarding the effectiveness of adenotonsillectomy in adults with PFAPA. Available reports suggest that surgical outcomes in adults may be less favorable than those observed in pediatric populations. Nevertheless, because PFAPA in adults typically does not resolve spontaneously, tonsillectomy has been proposed as a therapeutic option worth considering in selected cases [71]. Given the generally benign and self-limiting nature of PFAPA and the potential risks of surgery, adenotonsillectomy should be reserved for carefully selected patients with frequent or refractory episodes and substantial disease burden. Although tonsillectomy may reduce attacks in some patients, evidence remains limited by small trials, possible spontaneous remission, and reported recurrence after surgery. Thus, its potential benefits should be carefully weighed against surgical risks and considered on an individual basis, especially when repeated corticosteroid treatment is unsuitable or ineffective [63].

#### 8.2.4. Vitamin D

Vitamin D appears to play an immunomodulatory role in PFAPA by affecting T-cell proliferation and dendritic cell activity. Low or suboptimal serum vitamin D concentrations have been reported in a substantial proportion of children with PFAPA. Vitamin D supplementation with 400 IU daily for several months has been associated with a meaningful reduction in both the frequency and duration of typical PFAPA episodes [36].

In a recent cohort of 71 patients with PFAPA, 33.8% had vitamin D insufficiency (25(OH)D of 50–75 nmol/L) and 66.2% had vitamin D deficiency (levels of <50 nmol/L). Among those with insufficient levels, vitamin D supplementation (2000 IU/day) for 2–4 months was associated with a non-significant reduction in annual attack frequency and mean attack duration. In contrast, patients with vitamin D deficiency experienced a statistically significant decline in both attack frequency (from 7.4 ± 2.1 to 3.3 ± 2.4 episodes per year; *p* < 0.01) and duration (from 2.2 ± 1.6 to 1.3 ± 0.9 days; *p* = 0.04) following supplementation. A serum vitamin D threshold of 29.7 nmol/L was identified as the cut-off value associated with attack frequency [17]. While these findings are promising, they do not provide enough evidence to definitively state that vitamin D can treat or prevent PFAPA. Establishing clinical efficacy will require more rigorous research through RCTs and long-term studies involving much larger groups of patients.

#### 8.2.5. Probiotics

Probiotics are progressively being employed as a preventive strategy against infections and immune dysregulation flares. In a cohort of 20 pediatric PFAPA patients, adjunctive probiotic therapy was administered during the disease course using *Lactobacillus plantarum HEAL9* and *Lactobacillus paracasei* 8700:2, with a median treatment duration of 4.5 months. Nineteen of the 20 patients exhibited a reduction in attack frequency following probiotic initiation. The median number of PFAPA episodes over a 3-month period declined significantly from 3 to 1 (*p* < 0.001). Eight patients (40%) experienced complete remission of attacks during the first three months of the probiotic therapy. Among the 11 patients who continued to have at least one episode while receiving probiotics, 5 (45%) reported a marked reduction in attack severity [72].

In a multicenter study of 85 pediatric PFAPA patients, long-term prophylaxis with *Streptococcus salivarius K12 (SSK12)* was found to significantly reduce the clinical burdens. Over a median treatment period of six months, the frequency of febrile flares dropped by more than half, while individual episodes became shorter (from median 4 to 2 days, *p* < 0.001) and less intense with improving fever pattern. Beyond symptom relief, probiotics demonstrated a clear steroid-sparing effect, significantly lowering annual corticosteroid exposure and reducing the prevalence of hallmark symptoms like pharyngitis and oral aphthous ulcers [39].

Another recent retrospective larger analysis of 117 pediatric PFAPA patients demonstrated that six months of SSK12 prophylaxis significantly spaced the mean interval between febrile flares from 26.1 days to 70 days. Moreover, probiotics effectively reduced fever durations (falling from 4.1 to 3.3 days) and lower peak temperatures (decreasing from 39.8 °C to 39.1 °C), (all *p* values < 0.01). Furthermore, oral aphthosis, cervical lymphadenopathy, and pharyngotonsillitis have also improved, suggesting that probiotics improve the overall quality of life of PFAPA patients and potentially limit the need for pharmacological/surgical interventions [73]. While these results are promising, prospective RCTs are essential to further characterize the long-term efficacy and safety profile of probiotics in broader pediatric populations.

### 8.3. Other Experimental Therapies

Thalidomide may have an immunomodulatory effect and has been reported as a therapeutic option in refractory or persistent PFAPA syndrome, provided careful monitoring for adverse effects is ensured [74]. Montelukast has been reported in treatment of one adult PFAPA patient spacing febrile episodes [75]. Pidotimod has shown promising clinical benefit in pediatric patients with PFAPA. In a prospective, controlled, open-label study, high-dose pidotimod was found to be safe and effective in reducing the frequency and severity of PFAPA flares, while not altering the overall natural course of the disease [76]. Interestingly, an adolescent female patient with a childhood history of PFAPA later developed clinical features consistent with Behçet’s spectrum disorder, has been achieved remission with upadacitinib, suggesting that JAK inhibition may represent a rational therapeutic strategy in patients with overlapping PFAPA and Behçet’s spectrum features [77]. Overall, while emerging and experimental therapies show promising clinical benefits in selected PFAPA patients, their use remains preliminary and warrants validation through larger controlled studies to better define efficacy, optimal dosing, long-term safety, and appropriate patient selection.

The CARRA Working Group established predefined outcome criteria. The main endpoint was resolution of febrile episodes, classified as complete response (absence of fever over 3 months), partial response (decrease in total fever days within 3 months), or no response (unchanged or increased fever days during the same period). Each therapeutic strategy was recommended to be assessed over 3 consecutive febrile episodes before judging efficacy, with transition to an alternative approach if considered ineffective by the clinician and/or family. CARRA consensus treatment plans (CTPs) were established to provide a standardized framework for PFAPA management and to support comparison of different treatment approaches in clinical studies. They represent expert consensus rather than formal guidelines favoring one treatment strategy over another [14] (Table 3).

**Table 3:** Therapeutic approach guided by the CARRA consensus treatment plans [14].

Management Strategy	Description
Antipyretic arm	Supportive during febrile episodes such as paracetamol or NSAIDs
Corticosteroid arm	• Administration of prednisolone 1 mg/kg as a single dose within 24 h of fever onset (may need another dose on day 2 of fever *, then if interval between febrile episodes: • ≥3 weeks interval, continue the same regimen. • <2 weeks interval, change to another arm. • 2–3 weeks interval trial of 2 mg/kg prednisolone and according to response: ⮚ >3 weeks interval, continue with that dose ⮚ <3 weeks interval, change to another arm
Prophylactic arm	Colchicine (0.5–1.25 mg/day) or cimetidine (20–40 mg/kg/day, divided into 2 doses.
Surgical arm	Tonsillectomy/Adenotonsillectomy

* If fever recurs within 12–24 h of first prednisolone dose.

## 9. Prognosis

PFAPA is generally considered a benign, self-resolving condition. In a long-term follow-up cohort ranged from 12 to 21 years of children with PFAPA, approximately 85% achieved complete remission; the mean reported disease duration was approximately six years, although remission rates may vary across populations and follow-up periods. Febrile episodes typically decline in intensity, occur less frequently, and become shorter in duration over time. In individuals whose symptoms extend into adolescence, attacks usually become milder and less frequent compared with earlier stages of the disease. Growth and developmental outcomes remain normal, and significant long-term complications have rarely been reported [78]. In a large Turkish cohort of 466 PFAPA patients, factors associated with earlier remission included older age at disease onset, a positive family history of PFAPA, and absence of headache during flares [79].

**Future Research Directions:** Despite substantial progress in understanding PFAPA, several aspects of its pathogenesis and clinical management remain incompletely defined. Future research should prioritize the prospective validation of biomarkers that can distinguish PFAPA from monogenic autoinflammatory diseases and recurrent infections and predict disease activity or treatment response. Functional genomic studies should investigate the biological relevance of variants in genes such as *NLRP3*, *CARD8*, and *ALPK1*, while proteomic, transcriptomic, and immunometabolic approaches may help identify reproducible molecular signatures and biologically distinct PFAPA phenotypes. Longitudinal microbiome studies, ideally integrating microbial, immune, and metabolic data, are needed to determine whether reported microbial alterations contribute to disease pathogenesis or represent secondary changes. In parallel, mechanistic studies of dysregulated inflammatory pathways could identify candidate therapeutic targets for future clinical investigation, including pathway-specific immunomodulation in patients with severe or treatment-refractory disease. Multicenter prospective studies and randomized controlled trials should ultimately evaluate these emerging biomarkers and therapeutic targets using standardized clinical and biological outcomes, with particular attention to predictors of spontaneous remission, treatment response, and long-term disease course.

## 10. Conclusions

PFAPA is a clinically heterogeneous, self-limited autoinflammatory syndrome with an incompletely understood pathogenesis, particularly regarding its periodicity and localization to oropharyngeal lymphoid tissue and may overlap genetically and clinically with monogenic disorders such as FMF. Diagnosis remains challenging. Management should prioritize optimized medical therapy reserving surgical intervention for selected or refractory cases. Pediatricians should consider PFAPA during the initial assessment of children presenting with recurrent, stereotyped febrile episodes, particularly when accompanied by pharyngitis, aphthous stomatitis, or cervical adenitis, to ensure proper management and avoid unnecessary antibiotic use. Management should be individualized according to disease severity and burden. Corticosteroids and, in selected patients, tonsillectomy remain established treatment approaches, while colchicine may be considered for prophylaxis in selected cases. Evidence for IL-1 inhibitors, probiotics, vitamin D, and other emerging therapies remains limited, and these interventions should not be considered routine treatment without further supportive evidence. Further well-designed studies are required to refine classification criteria, elucidate the underlying pathophysiological mechanisms, and optimize evidence-based management strategies for PFAPA. A limitation of this study is that only English-language publications were included, which may have resulted in the omission of relevant studies published in other languages.

## Figures and Tables

**Table 1:** PFAPA diagnostic and classification criteria.

Criteria	Description
Marshall’s (1987) (Diagnostic criteria) [3]	- Age of onset generally early childhood - Regularly recurring febrile episodes - At least one of pharyngitis, aphthous ulcers and cervical lymphadenitis (in absence of URTI) - Asymptomatic between the attacks and although normal growth - Exclusion of cyclic neutropenia, immunodeficiency autoimmunity and other periodic fevers
Modified Marshall’s (1999) (Diagnostic criteria) [4]	- Age of onset typically <5 years old - Regularly recurring febrile episodes - At least one of pharyngitis, aphthous ulcers and cervical lymphadenitis (in absence of URTI) - Asymptomatic between the attacks and although normal growth - Exclusion of cyclic neutropenia
Cantarini et al. (2017) (Adult-Onset PFAPA classification criteria) [5]	- Age of onset at ≥16 years - Recurring febrile episodes - Pharyngitis or cervical lymphadenitis - High inflammatory markers during the attacks and asymptomatic in between - Exclusion of Infections, malignancy, and autoimmune/autoinflammatory diseases
Vanoni et al. (2018) (Classification criteria) [6]	- Age of onset < 6 years old - Fever episodes reach ≥38.5 °C, last 2–7 days, and regularly recur at least five times over a minimum of six months, with intervals between attacks not exceeding two months - At least one of pharyngitis, aphthous ulcers and cervical lymphadenitis - Asymptomatic between the attacks and although normal growth - Absence of infections, immunodeficiency and cyclic neutropenia and other causes of recurrent fever
New Eurofever/PRINTO (2019) (Classification criteria) [7]	At least 7 out of 8: - Pharyngotonsillitis - Febrile episodes last 3–6 days - Cervical lymphadenitis - Regular periodicity of the fever episodes - No diarrhea - No chest pain - No cutaneous rash - No arthritis

## Data Availability

No new data were created or analyzed in this study. Data sharing is not applicable to this article.

## Abbreviations

The following abbreviations are used in this manuscript:

BSDs	Behcet’s Spectrum Disorders
CAPS	Cryopyrin-Associated Periodic Syndrome
CARD8	Caspase recruitment domain-containing protein 8
CARRA	Childhood Arthritis and Rheumatology Association
CBC	Complete blood count
CD8+	Cluster of Differentiation 8
CRP	C-Reactive Protein
ESR	Erythrocyte Sedimentation Rate
FMF	Familial Mediterranean Fever
HA20	Haploinsufficiency of A20
HLA	Human Leukocyte Antigen
IFN-γ	Interferon- gamma
IL-1	Interleukin-1
IL-1β	Interleukin-1 beta
IQR	Interquartile Range
JAK	Janus Kinase
MAIDs	Mixed Autoinflammatory Disorders
MEFV	Mediterranean Fever
MKD/HIDS	Mevalonate Kinase Deficiency/Hyperimmunoglobulin D Syndrome
MRP8/14	Myeloid-Related Protein 8/14
NLRP3	NOD-like receptor family pyrin domain containing 3
NSAIDs	Nonsteroidal anti-inflammatory drugs
PFAPA	Periodic Fever, Aphthous stomatitis, Pharyngitis, and Adenitis
PRINTO	Paediatric Rheumatology International Trials Organisation
RAS	Recurrent aphthous stomatitis
RCT	Randomized controlled trial
S100	S100 calcium-binding protein
SAA	Serum Amyloid A
SAIDs	Systemic Autoinflammatory Disorders
SD	Standard Deviation
SSK12	Streptococcus salivarius K12
SURF	Syndrome of Undifferentiated Recurrent Fever
Th1	T-helper cells 1
Th17	T-helper cells 17
TNF	Tumor Necrosis Factor
TNFRSF1A	Tumor Necrosis Factor Receptor Superfamily Member 1A
TRAPS	TNF-receptor-associated periodic syndrome
VUS	Variants of Uncertain Significance

## References

[1] Førsvoll J., Kristoffersen E.K., Øymar K. (2013). Incidence, clinical characteristics and outcome in Norwegian children with periodic fever, aphthous stomatitis, pharyngitis and cervical adenitis syndrome: A population-based study. Acta Paediatr..

[2] Özaslan Z., Şen A., Uçar S.A., Çakan M., Sanisoğlu B., Kaya F., Otar Yener G., Demir F., Tanatar A., Özdel S. (2024). Exploring factors for predicting colchicine responsiveness in children with PFAPA. Eur. J. Pediatr..

[3] Marshall G.S., Edwards K.M., Butler J., Lawton A.R. (1987). Syndrome of periodic fever, pharyngitis, and aphthous stomatitis. J. Pediatr..

[4] Thomas K.T., Feder H.M., Lawton A.R., Edwards K.M. (1999). Periodic fever syndrome in children. J. Pediatr..

[5] Cantarini L., Vitale A., Sicignano L.L., Emmi G., Verrecchia E., Patisso I., Cerrito L., Fabiani C., Cevenini G., Frediani B. (2017). Diagnostic criteria for adult-onset periodic fever, aphthous stomatitis, pharyngitis, and cervical adenitis (PFAPA) syndrome. Front. Immunol..

[6] Vanoni F., Caorsi R., Aeby S., Cochard M., Antón J., Berg S., Brik R., Dolezalova P., Koné-Paut I., Neven B. (2018). Towards a new set of classification criteria for PFAPA syndrome. Pediatr. Rheumatol. Online J..

[7] Gattorno M., Hofer M., Federici S., Vanoni F., Bovis F., Aksentijevich I., Antón J., Arostegui J.I., Barron K., Ben-Chetrit E. (2019). Classification criteria for autoinflammatory recurrent fevers. Ann. Rheum. Dis..

[8] Wang A., Manthiram K., Dedeoglu F., Licameli G.R. (2021). Periodic fever, aphthous stomatitis, pharyngitis, and adenitis (PFAPA) syndrome: A review. World J. Otorhinolaryngol. Head Neck Surg..

[9] Anselmi F., Dusser P., Koné-Paut I. (2025). Periodic fever, aphthous stomatitis, pharyngitis, and cervical adenitis (PFAPA) syndrome in children—From pathogenesis to treatment strategies: A comprehensive review. Paediatr. Drugs.

[10] Labouret M., Elhani I., Cavelot S., Dingulu G., Jouret M., Nguyen A.T., Subervie A., Tavernier A.S., Vinit C., Hentgen V. (2025). Symptom course of PFAPA syndrome. Pediatr. Rheumatol. Online J..

[11] Michailou M., Perdikogianni C. (2025). Periodic fever, aphthous stomatitis, pharyngitis, and adenitis syndrome: An update. Children.

[12] Manthiram K. (2023). What is PFAPA syndrome? Genetic clues about the pathogenesis. Curr. Opin. Rheumatol..

[13] Amikishiyev S., Kalaycı T., Deniz R., Soltanova L., Sen B., Yalçınkaya Y., Esen B.A., Inanc M., Sahin A., Aday A. (2026). Phenotypes of patients with more than one autoinflammatory disease-associated gene variant: Overlapping and mixed autoinflammatory disorders. Rheumatology.

[14] Amarilyo G., Rothman D., Manthiram K., Edwards K.M., Li S.C., Marshall G.S., Yildirim-Toruner C., Haines K., Ferguson P.J., Lionetti G. (2020). Consensus treatment plans for periodic fever, aphthous stomatitis, pharyngitis, and adenitis syndrome (PFAPA): A framework to evaluate treatment responses from the Childhood Arthritis and Rheumatology Research Alliance (CARRA) PFAPA Work Group. Pediatr. Rheumatol. Online J..

[15] Asna Ashari K., Rezaei N. (2021). PFAPA (periodic fever, aphthous stomatitis, pharyngitis, and adenitis) syndrome: An overview of genetic background. Clin. Rheumatol..

[16] Rigante D., Calò L., Ciavarro A., Galli J. (2023). A potential partnership between genetics and the oral microbiome in children displaying periodic fever/aphthosis/pharyngitis/adenitis syndrome. Int. J. Mol. Sci..

[17] Öner N., Çelikel E., Tekin Z.E., Güngörer V., Tekgöz N., Sezer M., Karagöl C., Coşkun S., Kaplan M.M., Polat M.C. (2024). The effect of vitamin D supplementation on attacks in PFAPA syndrome patients with low vitamin D levels. Ir. J. Med. Sci..

[18] Berkun Y., Levy R., Hurwitz A., Meir-Harel M., Lidar M., Livneh A., Padeh S. (2011). The familial Mediterranean fever gene as a modifier of periodic fever, aphthous stomatitis, pharyngitis, and adenopathy syndrome. Semin. Arthritis Rheum..

[19] Kazanasmaz H., Gündoğmuş F., Eroğlu E.C., Kalyoncu M. (2026). Serum amyloid A as a predictive factor in PFAPA syndrome attacks. Med. Clin..

[20] Konte E.K., Haslak F., Yildiz M., Gucuyener N., Ulkersoy I., Gunalp A., Aslan E., Adrovic A., Sahin S., Barut K. (2023). Gray zone in the spectrum of autoinflammatory diseases: Familial Mediterranean fever accompanying periodic fever, aphthous stomatitis, pharyngitis, and adenitis syndrome: Single-center experience. Eur. J. Pediatr..

[21] Manthiram K., Preite S., Dedeoglu F., Demir S., Ozen S., Edwards K.M., Lapidus S., Katz A.E., Feder H.M., Genomic Ascertainment Cohort (2020). Common genetic susceptibility loci link PFAPA syndrome, Behçet’s disease, and recurrent aphthous stomatitis. Proc. Natl. Acad. Sci. USA.

[22] Dytrych P., Krol P., Kotrova M., Kuzilkova D., Hubacek P., Krol L., Katra R., Hrusak O., Kabelka Z., Dolezalova P. (2015). Polyclonal, newly derived T cells with low expression of inhibitory molecule PD-1 in tonsils define the phenotype of lymphocytes in children with periodic fever, aphthous stomatitis, pharyngitis, and adenitis (PFAPA) syndrome. Mol. Immunol..

[23] Kolly L., Busso N., von Scheven-Gete A., Bagnoud N., Moix I., Holzinger D., Simon G., Ives A., Guarda G., So A. (2013). Periodic fever, aphthous stomatitis, pharyngitis, cervical adenitis syndrome is linked to dysregulated monocyte IL-1β production. J. Allergy Clin. Immunol..

[24] Cheung M.S., Theodoropoulou K., Lugrin J., Martinon F., Busso N., Hofer M. (2017). Periodic fever with aphthous stomatitis, pharyngitis, and cervical adenitis syndrome is associated with a CARD8 variant unable to bind the NLRP3 inflammasome. J. Immunol..

[25] Pelagatti M.A., Meini A., Caorsi R., Cattalini M., Federici S., Zulian F., Calcagno G., Tommasini A., Bossi G., Sormani M.P. (2011). Long-term clinical profile of children with the low-penetrance R92Q mutation of the *TNFRSF1A* gene. Arthritis Rheum..

[26] Bens S., Zichner T., Stütz A.M., Caliebe A., Wagener R., Hoff K., Korbel J.O., von Bismarck P., Siebert R. (2014). *SPAG7* is a candidate gene for the periodic fever, aphthous stomatitis, pharyngitis, and adenopathy (PFAPA) syndrome. Genes Immun..

[27] Sangiorgi E., Azzarà A., Molinario C., Pietrobono R., Rigante D., Verrecchia E., Sicignano L.L., Genuardi M., Gurrieri F., Manna R. (2019). Rare missense variants in the *ALPK1* gene may predispose to periodic fever, aphthous stomatitis, pharyngitis, and adenitis (PFAPA) syndrome. Eur. J. Hum. Genet..

[28] Perko D., Debeljak M., Toplak N., Avčin T. (2015). Clinical features and genetic background of the periodic fever syndrome with aphthous stomatitis, pharyngitis, and adenitis: A single-center longitudinal study of 81 patients. Mediat. Inflamm..

[29] Di Gioia S.A., Bedoni N., von Scheven-Gête A., Vanoni F., Superti-Furga A., Hofer M., Rivolta C. (2015). Analysis of the genetic basis of periodic fever with aphthous stomatitis, pharyngitis, and cervical adenitis (PFAPA) syndrome. Sci. Rep..

[30] Kışla Ekinci R.M., Anlaş Ö., Özalp Ö. (2023). Utility of a targeted next-generation sequencing-based genetic screening panel in patients with periodic fever, aphthous stomatitis, pharyngitis, and adenitis syndrome. Arch. Rheumatol..

[31] Purrahman D., Poniatowski Ł.A., Wojdasiewicz P., Fathi M.R., Yousefi H., Lak E., Mahmoudian-Sani M.R. (2022). The role of inflammatory mediators in the pathogenesis of periodic fever, aphthous stomatitis, pharyngitis, and cervical adenitis (PFAPA) syndrome. Mol. Biol. Rep..

[32] Manthiram K., Ortega-Villa A.M., Lapidus S., Bowes M., Romeo T., Garguilo K., Failla L., Srinivasalu H., Mudd P., Ombrello A. (2025). Features associated with response to tonsillectomy in periodic fever, aphthous stomatitis, pharyngitis, and cervical adenitis syndrome in children. J. Pediatr..

[33] Førsvoll J., Janssen E.A., Møller I., Wathne N., Skaland I., Klos J., Kristoffersen E.K., Øymar K. (2015). Reduced number of CD8+ cells in tonsillar germinal centres in children with the periodic fever, aphthous stomatitis, pharyngitis, and cervical adenitis syndrome. Scand. J. Immunol..

[34] Mutlu F., Kasap M., Yaprak Bayrak B., Sarıhan M., Şahin N., Önal A., Akpınar G., Bayrak Y.E., Sönmez H.E. (2025). The first proteomics analysis of tonsils in patients with periodic fever, aphthous stomatitis, pharyngitis, and adenitis syndrome (PFAPA). Pediatr. Res..

[35] Rigante D., Manna R., Candelli M. (2025). Exploring the significance of vitamin D insufficiency in the periodic fever, aphthous stomatitis, pharyngitis, and cervical adenitis (PFAPA) syndrome: A single-center retrospective assessment during the decade 2014–2024. Intern. Emerg. Med..

[36] Stagi S., Bertini F., Rigante D., Falcini F. (2014). Vitamin D levels and effects of vitamin D replacement in children with periodic fever, aphthous stomatitis, pharyngitis, and cervical adenitis (PFAPA) syndrome. Int. J. Pediatr. Otorhinolaryngol..

[37] Galli J., Calò L., Posteraro B., Rossi G., Sterbini F.P., Paludetti G., Sanguinetti M. (2020). Pediatric oropharyngeal microbiome: Mapping in chronic tonsillitis and tonsillar hypertrophy. Int. J. Pediatr. Otorhinolaryngol..

[38] Tejesvi M.V., Uhari M., Tapiainen T., Pirttilä A.M., Suokas M., Lantto U., Koivunen P., Renko M. (2016). Tonsillar microbiota in children with PFAPA (periodic fever, aphthous stomatitis, pharyngitis, and adenitis) syndrome. Eur. J. Clin. Microbiol. Infect. Dis..

[39] La Torre F., Sota J., Insalaco A., Conti G., Del Giudice E., Lubrano R., Breda L., Maggio M.C., Civino A., Mastrorilli V. (2023). Preliminary data revealing efficacy of *Streptococcus salivarius* K12 (SSK12) in periodic fever, aphthous stomatitis, pharyngitis, and cervical adenitis (PFAPA) syndrome: A multicenter study from the AIDA Network PFAPA syndrome registry. Front. Med..

[40] Batu E.D. (2019). Periodic fever, aphthous stomatitis, pharyngitis, and cervical adenitis (PFAPA) syndrome: Main features and an algorithm for clinical practice. Rheumatol. Int..

[41] Lantto U., Koivunen P., Tapiainen T., Renko M. (2016). Long-term outcome of classic and incomplete PFAPA (periodic fever, aphthous stomatitis, pharyngitis, and adenitis) syndrome after tonsillectomy. J. Pediatr..

[42] Rigante D., Vitale A., Natale M.F., Lopalco G., Andreozzi L., Frediani B., D’Errico F., Iannone F., Cantarini L. (2017). A comprehensive comparison between pediatric and adult patients with periodic fever, aphthous stomatitis, pharyngitis, and cervical adenopathy (PFAPA) syndrome. Clin. Rheumatol..

[43] Marcuzzi A., Piscianz E., Kleiner G., Tommasini A., Severini G.M., Monasta L., Crovella S. (2013). Clinical genetic testing of periodic fever syndromes. Biomed. Res. Int..

[44] Rydenman K., Berg S., Karlsson-Bengtsson A., Fasth A., Wekell P. (2024). Antibiotic prescriptions to children with periodic fever, aphthous stomatitis, pharyngitis, and cervical adenitis. Acta Paediatr..

[45] Adrovic A., Sahin S., Barut K., Kasapcopur O. (2019). Familial Mediterranean fever and periodic fever, aphthous stomatitis, pharyngitis, and adenitis (PFAPA) syndrome: Shared features and main differences. Rheumatol. Int..

[46] Gardner N.J. (2023). PFAPA syndrome in children. JAAPA.

[47] Parmaksız G., Noyan Z.A. (2023). Can RDW be used as a screening test for subclinical inflammation in children with FMF? Is RDW related to *MEFV* gene mutations?. Clin. Rheumatol..

[48] Aktaş B., Gümüş D., Tunalı A., Kunter Z., Adrovic A. (2022). Mevalonate kinase deficiency/hyperimmunoglobulin D syndrome (MVK/HIDS) in the differential diagnosis of periodic fever, aphthous stomatitis, pharyngitis, and cervical adenitis (PFAPA) syndrome and familial Mediterranean fever (FMF): A case report. Turk. Arch. Pediatr..

[49] Jo K.J., Park S.E., Cheon C.K., Oh S.H., Kim S.H. (2023). Haploinsufficiency A20 misdiagnosed as PFAPA (periodic fever, aphthous stomatitis, pharyngitis, and cervical adenitis) syndrome with Kikuchi disease. Clin. Exp. Pediatr..

[50] Palmeri S., Penco F., Bertoni A., Bustaffa M., Matucci-Cerinic C., Papa R., Drago E., Caorsi R., Corcione A., Bocca P. (2024). Pyrin inflammasome activation defines colchicine-responsive SURF patients from FMF and other recurrent fevers. J. Clin. Immunol..

[51] Papa R., Penco F., Volpi S., Sutera D., Caorsi R., Gattorno M. (2021). Syndrome of undifferentiated recurrent fever (SURF): An emerging group of autoinflammatory recurrent fevers. J. Clin. Med..

[52] Güngörer V., Ünal D., Çakan M., Ayduran S., Gül Ü., Zora H.K., Öner N., Köker O., Uçak K., Şahin N. (2025). Syndrome of undifferentiated recurrent fever (SURF): A multicenter real-world experience from Türkiye. Clin. Rheumatol..

[53] Gürel Bedir A., Kılıç S.S. (2025). Clinical characteristics and quality of life in children with PFAPA syndrome and Behçet’s disease. Rheumatol. Adv. Pract..

[54] Ter Haar N., Lachmann H., Özen S., Woo P., Uziel Y., Modesto C., Koné-Paut I., Cantarini L., Insalaco A., Neven B. (2013). Treatment of autoinflammatory diseases: Results from the Eurofever Registry and a literature review. Ann. Rheum. Dis..

[55] Cattalini M., Soliani M., Rigante D., Lopalco G., Iannone F., Galeazzi M., Cantarini L. (2015). Basic characteristics of adults with periodic fever, aphthous stomatitis, pharyngitis, and adenopathy syndrome in comparison with the typical pediatric expression of disease. Mediat. Inflamm..

[56] Yazgan H., Gültekin E., Yazıcılar O., Sagun Ö.F., Uzun L. (2012). Comparison of conventional and low-dose steroid in the treatment of PFAPA syndrome: Preliminary study. Int. J. Pediatr. Otorhinolaryngol..

[57] Soylu A., Yıldız G., Torun Bayram M., Kavukçu S. (2021). IL-1β blockade in periodic fever, aphthous stomatitis, pharyngitis, and cervical adenitis (PFAPA) syndrome: Case-based review. Rheumatol. Int..

[58] Stojanov S., Lapidus S., Chitkara P., Feder H., Salazar J.C., Fleisher T.A., Brown M.R., Edwards K.M., Ward M.M., Colbert R.A. (2011). Periodic fever, aphthous stomatitis, pharyngitis, and adenitis (PFAPA) is a disorder of innate immunity and Th1 activation responsive to IL-1 blockade. Proc. Natl. Acad. Sci. USA.

[59] Gunes M., Cekic S., Kilic S.S. (2017). Is colchicine more effective to prevent periodic fever, aphthous stomatitis, pharyngitis and cervical adenitis episodes in Mediterranean fever gene variants?. Pediatr. Int..

[60] Otar Yener G., Aktaş İ., Altıntaş Meşe C., Çakan M. (2023). Does having *MEFV* gene sequence variants affect the clinical course and colchicine response in children with PFAPA syndrome?. Eur. J. Pediatr..

[61] Koru L., Erten E.S.Y., Dursun H.K., Dizman E.N., Kaya F., Kucuk E., Aydin Z., Balci M.O., Ozturk K., Haslak F. (2025). Colchicine treatment in PFAPA: How long should we wait for the clinical response?. Eur. J. Pediatr..

[62] Sönmez H.E., Batu E.D., Bilginer Y., Özen S. (2017). Discontinuing colchicine in symptomatic carriers for *MEFV* (*Mediterranean FeVer*) variants. Clin. Rheumatol..

[63] Vanoni F., Theodoropoulou K., Hofer M. (2016). PFAPA syndrome: A review on treatment and outcome. Pediatr. Rheumatol. Online J..

[64] Raeeskarami S.R., Sadeghi P., Vahedi M., Asna Ashari K., Mousavi T.M., Ziaee V. (2022). Colchicine versus cimetidine: The better choice for periodic fever, aphthous stomatitis, pharyngitis, adenitis (PFAPA) syndrome prophylaxis, and the role of *MEFV* gene mutations. Pediatr. Rheumatol. Online J..

[65] Renko M., Salo E., Putto-Laurila A., Saxen H., Mattila P.S., Luotonen J., Ruuskanen O., Uhari M. (2007). A randomized, controlled trial of tonsillectomy in periodic fever, aphthous stomatitis, pharyngitis, and adenitis syndrome. J. Pediatr..

[66] Garavello W., Romagnoli M., Gaini R.M. (2009). Effectiveness of adenotonsillectomy in PFAPA syndrome: A randomized study. J. Pediatr..

[67] Lantto U., Tapiainen T., Pokka T., Koivunen P., Helminen M., Piitulainen J., Rekola J., Uhari M., Renko M. (2024). Tonsillotomy for periodic fever syndrome: A randomized and controlled trial. Laryngoscope.

[68] Moberg T., Rydenman K., Berg S., Fasth A., Wekell P. (2025). Long-term symptoms in periodic fever, aphthous stomatitis, pharyngitis, and cervical adenitis syndrome after tonsillectomy. J. Pediatr..

[69] Cavalcante L.F.F., Costa G., Battistuta S.M., Makabe P.F., Bueno I.S.F., Yuamoto B., Vilela F.E.G., Sáfadi M.A.P. (2025). Surgical management for periodic fever, aphthous stomatitis, pharyngitis and cervical adenitis (PFAPA) syndrome: A systematic review and meta-analysis. J. Pediatr..

[70] Noy R., Barzilai R., Cohen J.T., Gordin A., Zur K.B. (2025). Surgical Treatments for Periodic Fever, Aphthous Stomatitis, Pharyngitis, and Adenitis Syndrome: Systematic Review and Network Meta-analysis. Otolaryngol. Head Neck Surg..

[71] Gaggiano C., Rigante D., Sota J., Grosso S., Cantarini L. (2019). Treatment options for periodic fever, aphthous stomatitis, pharyngitis, and cervical adenitis (PFAPA) syndrome in children and adults: A narrative review. Clin. Rheumatol..

[72] Batu E.D., Kaya Akca U., Basaran O., Bilginer Y., Özen S. (2022). Probiotic use in the prophylaxis of periodic fever, aphthous stomatitis, pharyngitis, and adenitis (PFAPA) syndrome: A retrospective cohort study. Rheumatol. Int..

[73] Spagnolo A., Mileto V., Civino A., Maggio M.C., Risso P., Sestito S., Gallizzi R. (2024). Use of *Streptococcus salivarius* K12 in a cohort of PFAPA patients. Front. Pediatr..

[74] Marque M., Guillot B., Bessis D. (2007). Thalidomide for treatment of PFAPA syndrome. Oral Surg. Oral Med. Oral Pathol. Oral Radiol. Endod..

[75] Abolghasemi S., Atashi H.A., Paydar-Tali E., Olya M., Zaferani-Arani H. (2019). The first case of adult-onset periodic fever, aphthous stomatitis, pharyngitis, and adenitis syndrome with splenomegaly in Iran. Casp. J. Intern. Med..

[76] Manti S., Filosco F., Parisi G.F., Finocchiaro G.G., Papale M., Giugno A., Barone P., Leonardi S. (2020). Proposal for a new therapeutic high dosage of pidotimod in children with periodic fever, aphthous stomatitis, pharyngitis, adenitis (PFAPA) syndrome: A randomized controlled study. Ital. J. Pediatr..

[77] Oka H., Sumitomo S., Yamashita D., Ohmura K. (2025). Case report: Successful remission with upadacitinib in Behçet’s syndrome following a history of PFAPA syndrome. Int. J. Rheum. Dis..

[78] Wurster V.M., Carlucci J.G., Feder H.M., Edwards K.M. (2011). Long-term follow-up of children with periodic fever, aphthous stomatitis, pharyngitis, and cervical adenitis syndrome. J. Pediatr..

[79] Yildiz M., Haslak F., Adrovic A., Gucuyener N., Ulkersoy I., Koker O., Sahin S., Unlu G., Barut K., Kasapcopur O. (2021). Independent risk factors for resolution of periodic fever, aphthous stomatitis, pharyngitis, and adenitis syndrome within 4 years after the disease onset. Clin. Rheumatol..

